# Supplementary material for: In search of a “vocabulary for recreation”: Leisure-time physical activity among humanitarian migrants in regional Australia
Source: PLoS One. 2020 Oct 14;15(10):e0239747. doi: 10.1371/journal.pone.0239747 (PMC7556461; doi:10.1371/journal.pone.0239747)
Supplement: S1 File — (DOCX) [file pone.0239747.s001.docx]

**S1 File. Focus group schedule**

Section 1: Preamble

Focus group participants to be seated in a circle, or semi-circle. Focus group leader will briefly introduce the purpose and context of the study and focus group discussion topics.

Introductions and “ice breaker” - Ask each participant to:

1. Briefly introduce themselves, their country of origin and how long they’ve been in Australia. Also the language spoken at home, their religion and what they do for work (if applicable).

Brief conversation about leisure, what it is and our focus on leisure-time physical activity.

1. Choose a word or a phrase that best describes your leisure today.

Relevant issues about leisure and settlement might flow from group sharing.

Section 2: Main focus

| **Main question** | **Possible probes** |
| --- | --- |
| **Theme 1** | |
| Views on leisure | What are your views on exercise as a leisure-time physical activity? |
|  | Do you enjoy sport, exercise or any other physical activity? What is it about this activity that you enjoy? |
|  | Any other form of leisure that you enjoy/prefer? |
| **Theme 2** | |
| Changes | What were your PA habits in your country of origin? Have they changed? How? Why? |
|  | What did you do for leisure in your country of origin? Do you still do it here? If not, why not? |
| **Theme 3** | |
| Participation | What are your current patterns of PA participation? |
|  | Do you tend to do these activities on your own or with others? Which way do you prefer? Does it match with your current practice? If not, why not? |
|  | What is your main motivation for doing whatever leisure activity you like doing? |
| **Theme 4** | |
| Barriers | What are the main barriers for participating in PA in Coffs Harbour? How can they be overcome? |
|  | Are there any activities that you like doing that you can’t or just don’t do anymore? If yes, why? |
| **Theme 5** | |
| Other forms of PA | Do you exercise as part of your work? |
|  | Do you exercise as part of commuting (i.e. walk/ride to work)? |
