# Supplementary material for: In search of a “vocabulary for recreation”: Leisure-time physical activity among humanitarian migrants in regional Australia
Source: PLoS One. 2020 Oct 14;15(10):e0239747. doi: 10.1371/journal.pone.0239747 (PMC7556461; doi:10.1371/journal.pone.0239747)
Supplement: S2 File — (PDF) [file pone.0239747.s002.pdf]

WESTERN SYDNEY UNIVERSITY

Date: ________________

Kit number: ___________

Section 1 - Physical Activity Participation

The questions below are about the time you spent being physically active in the last 7 days. They include questions about activities you do at work, as part of your house and yard work, to get from place to place, and in your spare time for recreation, exercise or sport. Please answer each question even if you do not consider yourself to be an active person.

In answering the following questions,

- **Vigorous** physical activities refer to activities that take hard physical effort and make you breathe much harder than normal
- **Moderate** activities refer to activities that take moderate physical effort and make you breathe somewhat harder than normal.

1. During the **last 7 days**, on how many days did you do **vigorous** physical activities like heavy lifting, digging, aerobics or fast bicycling? Think about only those physical activities that you did for at least 10 minutes at a time.

________ **days** ❑ None → **Skip to question 3**

2. How much time in total did you usually spend on one of those days doing **vigorous** physical activities?

________ **hours** ________ **minutes**

3. Again, think about only those physical activities that you did for at least 10 minutes at a time. During the **last 7 days**, on how many days did you do **moderate** physical activities like carrying light loads or bicycling at a regular pace? Please do not include walking.

________ **days per week** ❑ None → **Skip to question 5**

4. How much time in total did you usually spend on one of those days doing **moderate** physical activities?

________ **hours** ________ **minutes**

5. During the **last 7 days**, on how many days did you **walk** for at least 10 minutes at a time? This includes walking at work and at home, walking to travel from place to place, and any other walking that you did solely for recreation, sport, exercise or leisure.

________ **days per week** ❑ None → **Skip to question 7**

6. How much time in total did you usually spend **walking** on one of those days?

________ **hours** ________ **minutes**

Date: ________________

Kit number: ___________

The next question is about the time you spent sitting on weekdays while at work, at home, while doing course work and during leisure time. This includes time spent sitting at a desk, visiting friends, reading traveling on a bus or sitting or lying down to watch television.

7. During the **last 7 days**, how much time did you usually spend **sitting** on a **weekday**?

_______ **hours** _______ **minutes**

8. Are any of the factors below important in preventing you from participating in physical activity during your leisure time?

	Very Important	Somewhat Important	Not at all Important
The cost is too high	❑1	❑2	❑3
I don't know where to go	❑1	❑2	❑3
I don't have time/I am too busy	❑1	❑2	❑3
I don't have people to go with	❑1	❑2	❑3
I am afraid of getting hurt	❑1	❑2	❑3
I (or family members) have been in poor health	❑1	❑2	❑3
I am not interested in/don't enjoy doing physical activity	❑1	❑2	❑3
I don't speak English well enough	❑1	❑2	❑3
I don't feel comfortable around Australians yet	❑1	❑2	❑3
It is not part of my culture to exercise for fun or fitness	❑1	❑2	❑3
My religious beliefs do not support exercising for fun or fitness	❑1	❑2	❑3
I am concerned about being racially abused	❑1	❑2	❑3
I don't feel safe	❑1	❑2	❑3
I feel too tired	❑1	❑2	❑3
I have an injury/disease that prevents me from going	❑1	❑2	❑3
I feel too old	❑1	❑2	❑3

9. Please list below which activities you enjoy doing the most, and this time it can be any leisure activity (e.g. walking for leisure, dancing, singing, crafts, painting, gardening, swimming, bike riding, etc.):

____

____

____

10. Are there any physical activities that you participated in regularly BEFORE YOUR ARRIVAL IN AUSTRALIA?

❑1 Yes ❑2 No → **Skip to question 12**

11. If you answered YES to question 10, please list up to 2 such activities:

List activity	Do you still participate in this activity?	If not, why not?
1. ______________________	❑1 Yes ❑2 No	______________________
2. ______________________	❑1 Yes ❑2 No	______________________

12. Are there any physical activities that you now participate in SINCE ARRIVING IN AUSTRALIA?

❑1 Yes ❑2 No → **Skip to question 14**

13. If you answered YES to question 12, please list up to 2 new physical activities you now participate in.

Activity 1____

Activity 2____

Section 2 – About You and Your Health

14. What is your date of birth? _____/_____/_____

15. What is your gender? ❑1 Female ❑2 Male

16. How tall are you without shoes? ______ cm OR ___•___ feet

17. About how much do you weigh? ______ kg OR ______ lbs

18. What year did you first come to live in Australia? _______

19. Do you have children?

❑1 Yes. How many? ____________________ ❑2 No

20. How many people live in your household (including you)? ______

21. How many of them are 18 and over (including you)? ______

22. In which country were you born?

❑$_1$ Afghanistan	❑$_6$ Iraq
❑$_2$ Burma	❑$_7$ Liberia
❑$_3$ Burundi	❑$_8$ Sierra Leone
❑$_4$ Congo	❑$_9$ Sudan
❑$_5$ Ivory Coast	❑$_{10}$ Other: ________________________________

23. What is your religion?

❑$_1$ Catholicism	❑$_5$ Islam
❑$_2$ Anglicanism	❑$_6$ Hinduism
❑$_3$ Other Christianism	❑$_7$ Judaism
❑$_4$ Buddhism	❑$_8$ Other: ________________________________

24. What is the highest qualification you have completed?

❑$_1$ No school certificate or other qualifications
❑$_2$ School or intermediate certificate (or equivalent)
❑$_3$ Higher school or leaving certificate (or equivalent)
❑$_4$ Trade/apprenticeship (e.g. hairdresser, chef)
❑$_5$ Certificate/diploma (e.g. child care, technician)
❑$_6$ University degree or higher

25. What best describes your current housing?

❑$_1$ House	❑$_5$ Mobile home
❑$_2$ Hostel for the aged	❑$_6$ Retirement village, self-care unit
❑$_3$ Nursing home	❑$_7$ House on farm
❑$_4$ Flat, unit, apartment	❑$_8$ Other

26. What is your current work status:

❑1 In full time paid work	❑6 Self-employed
❑2 In part time paid work	❑7 Doing unpaid work
❑3 Completely retired/pensioner	❑8 Studying
❑4 Partially retired	❑9 Looking after home/family
❑5 Disabled/sick	❑10 Unemployed
	❑11 Other

27. What is your usual yearly HOUSEHOLD income before tax, from all sources? (please include benefits, pensions, superannuation, etc.)

❑1 less than $5,000 per year	❑5 $30,000-$39,999 per year
❑2 $5,000-$9,999 per year	❑6 $40,000-$49,999 per year
❑3 $10,000-$19,999 per year	❑7 $50,000-$69,999 per year
❑4 $20,000-$29,999 per year	❑8 $70,000 or more per year
	❑9 I would rather not answer this question

28. What best describes your current situation:

❑1 Single	❑4 Divorced
❑2 Married	❑5 De facto/living with a partner
❑3 Widow	❑6 Separated

29. Have you ever been a regular smoker?

❑1 Yes ❑2 No → **Skip to 34**

30. How old were you when you started smoking regularly? ______ years old

31. Are you a regular smoker now?

❑1 Yes → **Skip to 33** ❑2 No

32. How old were you when you stopped smoking regularly? ______ years old

33. About how much do you or did you smoke on average each day? ______ cigarettes;pipes;cigars/day

34. About how many alcoholic drinks do you have each week? (one drink = a glass of wine, middy of beer or nip of spirits; put 0 if you do not drink or have less than one drink each week) ______ drinks/week

35. On how many days each week do you usually drink alcohol? ______ days each week

36. Do you regularly need help with daily tasks because of long-term illness or disability? (e.g. personal care, getting around, preparing meals) ❑1 Yes ❑2 No

37. Does your health now LIMIT YOU in any of the following activities?

	Yes, limited a lot	Yes, limited a little	No, not limited at all
VIGOROUS activities (e.g. running, strenuous sports)	❑1	❑2	❑3
MODERATE activities (e.g. pushing a vacuum cleaner, cycling at a constant pace)	❑1	❑2	❑3
Lifting or carrying shopping	❑1	❑2	❑3
Climbing several flights of stairs	❑1	❑2	❑3
Climbing one flight of stair	❑1	❑2	❑3
Walking one kilometre	❑1	❑2	❑3
Waling half a kilometre	❑1	❑2	❑3
Walking 100 metres	❑1	❑2	❑3
Bending, kneeling or stooping	❑1	❑2	❑3
Bathing or dressing yourself	❑1	❑2	❑3

38. During the past 4 weeks, about how often did you feel:

	None of the time	A little of the time	Some of the time	Most of the time	All of the time
tired out for no good reason?	❑1	❑2	❑3	❑4	❑5
nervous?	❑1	❑2	❑3	❑4	❑5
so nervous that nothing could calm you down?	❑1	❑2	❑3	❑4	❑5
hopeless?	❑1	❑2	❑3	❑4	❑5
restless or fidgety?	❑1	❑2	❑3	❑4	❑5
so restless that you could not sit still?	❑1	❑2	❑3	❑4	❑5
depressed?	❑1	❑2	❑3	❑4	❑5
that everything was an effort?	❑1	❑2	❑3	❑4	❑5
So sad that nothing could cheer you up?	❑1	❑2	❑3	❑4	❑5
Worthless?	❑1	❑2	❑3	❑4	❑5

39. During the past 4 weeks, have you had any of the following problems with your work or daily activities because of any emotional problems (such as being depressed or anxious)?

	Yes	No
Cut down on the amount of time you spent on work or other activities	❑$_1$	❑$_2$
Achieved less than you would have liked to	❑$_1$	❑$_2$
Did work or other activities less carefully than usual	❑$_1$	❑$_2$

40. Has a doctor EVER told you that you have the following health conditions (if YES, please cross the box and give your age when the condition was first found):

	**YES**	**Age when condition was first found**
Heart disease	❑$_1$	______ age
High blood pressure	❑$_2$	______ age
Stroke	❑$_3$	______ age
Diabetes	❑$_4$	______ age
Blood clot (thrombosis)	❑$_5$	______ age
Asthma	❑$_6$	______ age
Depression	❑$_7$	______ age
Anxiety	❑$_8$	______ age
Post-traumatic stress disorder	❑$_9$	______ age
Chronic pain	❑$_{10}$	______ age
None of these	❑$_{11}$	______ age

41. In general, how would you rate your:

	**Excellent**	**Very good**	**Good**	**Fair**	**Poor**
overall health?	❑$_1$	❑$_2$	❑$_3$	❑$_4$	❑$_5$
quality of life?	❑$_1$	❑$_2$	❑$_3$	❑$_4$	❑$_5$

42. In the last month have you been treated for the following health conditions (if YES, please cross the box and give your age when the treatment started):

	YES	Age started treatment
Heart disease	❑1	______ age
High blood pressure	❑2	______ age
High blood cholesterol	❑3	______ age
Osteoarthritis	❑4	______ age
Osteoporosis or low bone density	❑5	______ age
Depression	❑6	______ age
Post-traumatic stress disorder	❑7	______ age
Chronic pain	❑8	______ age
Anxiety	❑9	______ age
None of these	❑10	______ age

Section 3 – About Your Personal Network

We would like you to answer the following questions thinking about the people you know living in Coffs Harbour.

43. How many people you know who live in Coffs Harbour you know by name and who also know you by name? ______

44. Of those, how many did you meet, or talk by phone or via social media in the last 30 days? ______

45. Of those you have met or talked to in the last 30 days, how many would you invite to take part in this research? ______

46. What is your relationship with the person who invited you to participate in this survey?

❑1 Lives in the same house as me	❑5 Close friend
❑2 Parent	❑6 Acquaintance
❑3 Sibling	❑7 Work colleague
❑4 Relative	❑8 Other: ________________________________

47. Of the people you know by name and who know you by name also, how many live in each of the neighbourhoods below? (approximate numbers are fine). Please tick the box indicating which neighbourhood you live in.

	Number of people I know who live here	I live here
Coffs Harbour CBD, Park Beach and Diggers Beach	______	❑1
Sawtell, Toormina, Boambee and Bonville	______	❑2
Orara, Karangi and Coramba	______	❑3
Korora, Sapphire and Monee	______	❑4
Emerald Beach, Sandy Beach and Woolgoolga	______	❑5

Thank you for your time!

If you have any further comments to contribute to this research, please feel free to use the space provided below. Your comments will be most appreciated and will add to the analysis of the data collected through this survey.

____

____

____

____

____

____

____

____
