# Supplementary material for: In search of a “vocabulary for recreation”: Leisure-time physical activity among humanitarian migrants in regional Australia
Source: PLoS One. 2020 Oct 14;15(10):e0239747. doi: 10.1371/journal.pone.0239747 (PMC7556461; doi:10.1371/journal.pone.0239747)
Supplement: S3 File — (DOCX) [file pone.0239747.s003.docx]

**S3 File. Interview schedule**

Section 1: Preamble

Interviewer will briefly introduce the purpose and context of the study and interview discussion topics.

Introduction and “ice breaker” - Ask the participant to:

1. Briefly introduce themselves, their country of origin and how long they’ve been in Australia. Also the language spoken at home, their religion and what they do for work (if applicable).

Brief conversation about leisure, what it is and our focus on leisure-time physical activity.

Section 2: Main focus

| **Main themes** | **Possible probes** |
| --- | --- |
| **Theme 1** | |
| Views on leisure | What are your views on exercise as a leisure-time physical activity? |
|  | Do you enjoy sport, exercise or any other physical activity? What is it about this activity that you enjoy? |
|  | Any other form of leisure that you enjoy/prefer? |
| **Theme 2** | |
| Changes | What were your PA habits in your country of origin? Have they changed? How? Why? |
|  | What did you do for leisure in your country of origin? Do you still do it here? If not, why not? |
|  | If your leisure has changed, how do you feel about it? |
|  | Are there any new leisure activities that you engage in here in Australia? If so, what are they? And why did you start to engage in it/them? |
| **Theme 3** | |
| Participation | What are your current patterns of PA participation? |
|  | Do you tend to do these activities on your own or with others? Which way do you prefer? Does it match with your current practice? If not, why not? |
|  | What is your main motivation for doing whatever leisure activity you like doing? |
| **Theme 4** | |
| Barriers | What are the main barriers for participating in PA in Coffs Harbour? How can they be overcome? |
|  | Are there any activities that you like doing that you can’t or just don’t do anymore? If yes, why? |
| **Theme 5** | |
| Other forms of PA | Do you exercise as part of your work? |
|  | Do you exercise as part of commuting (i.e. walk/ride to work)? |
| **Theme 6** | |
| Health | Do you consider yourself healthy? Why or why not? |
|  | How has immigration impacted on your health (both physical and mental)? |
|  | Do you feel leisure time PA or other activities help with your well-being? Why or why not? |
| **Theme 7** | |
| Support | Do you feel you have a support network in Coffs Harbour already? If so, who are they? |
|  | What sort of programs would or do support your health and well-being in Coffs Harbour? Are any of them related to leisure? |
