# Supplementary material for: In search of a “vocabulary for recreation”: Leisure-time physical activity among humanitarian migrants in regional Australia
Source: PLoS One. 2020 Oct 14;15(10):e0239747. doi: 10.1371/journal.pone.0239747 (PMC7556461; doi:10.1371/journal.pone.0239747)
Supplement: S1 Table — (DOCX) [file pone.0239747.s004.docx]

**S1 Table. Demographic characteristics**

| Variable |  | % (CI 95%) | Mean (CI 95%) |
| --- | --- | --- | --- |
| Gender | Female | 56.9 (48.4-65.3) |  |
|  | Male | 43.1 (33.4-52.8) |  |
| Number of people in the household |  |  | 4.6 (4.4-4.9) |
| Number of people in the household over 18yrs old |  |  | 2.7 (2.5-2.8) |
| Number of Children |  |  | 2.6 (2.3-2.9) |
| Religion | Catholicism | 8.6 (0.0-20.9) |  |
|  | Anglicanism | 1.3 (0.0-14.1) |  |
|  | Other Christian | 44.4 (34.8-54) |  |
|  | Buddhism | 0.9 (0.0-13.7) |  |
|  | Islam | 16.8 (5.1-28.5) |  |
|  | Yazidism | 28.0 (17.1-38.9) |  |
| Education | No schooling | 28.9 (18.0-39.7) |  |
|  | Primary or intermediate | 23.7 (12.5-34.9) |  |
|  | High school | 27.6 (16.6-38.5) |  |
|  | Trade/apprenticeship | 0.9 (0.0-13.7) |  |
|  | Certificate/diploma | 10.3 (0.0-22.5) |  |
|  | University or higher | 8.6 (0.0-20.9) |  |
| Housing | House | 55.6 (47-64.2) |  |
|  | Hostel for the aged | 0.0 (0.0-0.0) |  |
|  | Nursing home | 0.4 (0.0-13.3) |  |
|  | Apartment | 42.2 (32.5-52.0) |  |
|  | Mobile home | 0.0 (0.0-0.0) |  |
|  | Retirement village | 0.4 (0.0-13.3) |  |
|  | House on farm | 0.4 (0.0-13.3) |  |
|  | Other | 0.9 (0.0-13.7) |  |
| Employment | Full-time | 6.9 (0.0-19.4) |  |
|  | Part-time | 9.1 (0.0-21.4) |  |
|  | Completely retired | 1.3 (0.0-14.1) |  |
|  | Partially retired | 0.0 (0.0-0.0) |  |
|  | Disabled/sick | 0.9 (0.0-13.7) |  |
|  | Self-employed | 1.3 (0.0-14.1) |  |
|  | Unpaid work | 0.4 (0.0-13.3) |  |
|  | Studying | 51.9 (43-60.9) |  |
|  | Looking after home | 4.8 (0.0-17.3) |  |
|  | Unemployed | 13.9 (1.9-25.8) |  |
|  | Casual work | 9.5 (0.0-21.8) |  |
| Marital Status | Single | 35.8 (25.5-46.1) |  |
|  | Married | 51.7 (42.8-60.7) |  |
|  | Widow | 7.8 (0-20.1) |  |
|  | Divorced | 0.9 (0-13.7) |  |
|  | Living with a partner | 1.3 (0-14.1) |  |
|  | Separated | 2.6 (0-15.3) |  |
| Age | 18-19 | 8.3 (0.0-20.6) |  |
|  | 20-29 | 26.1 (15.0-37.2) |  |
|  | 30-39 | 25.2 (14.0-36.4) |  |
|  | 40-49 | 22.6 (11.2-34.0) |  |
|  | 50+ | 17.8 (6.1-29.5) |  |
| Length of arrival in Australia (in years) |  |  | 3.6 (3.1-4.1) |
|  | Up to 2 years | 47.4 (38.1-56.7) |  |
|  | 2-4 years | 19.0 (7.4-30.5) |  |
|  | 4-6 years | 9.9 (0.0-22.1) |  |
|  | 6-8 years | 9.1 (0.0-21.3) |  |
|  | More than 8 years | 14.6 (2.8-26.5) |  |
| Country of origin | Democratic Republic of Congo | 15.6 (3.7-27.4) |  |
|  | Syria | 15.2 (3.3-27.0) |  |
|  | Burma/Myanmar | 12.6 (0.5-24.6) |  |
|  | Iraq | 12.6 (0.5-24.6) |  |
|  | Afghanistan | 10.0 (0.0-22.2) |  |
|  | Ethiopia | 7.4 (0.0-19.8) |  |
|  | Togo | 6.1 (0.0-18.6) |  |
|  | Liberia | 5.2 (0.0-17.8) |  |
|  | Iran | 3.9 (0.0-16.5) |  |
|  | South Sudan | 3.5 (0.0-16.1) |  |
|  | Other | 7.8 (0.0-20.1) |  |
