# Supplementary material for: In search of a “vocabulary for recreation”: Leisure-time physical activity among humanitarian migrants in regional Australia
Source: PLoS One. 2020 Oct 14;15(10):e0239747. doi: 10.1371/journal.pone.0239747 (PMC7556461; doi:10.1371/journal.pone.0239747)
Supplement: S2 Table — (DOCX) [file pone.0239747.s005.docx]

**S2 Table. Health Indicators**

| **Variable** | **Level** | **Estimate** | **95% CI** |
| --- | --- | --- | --- |
| Smoking | Ever been regular | 7.8 | 0.00 - 20.12 |
|  | Currently regular | 4.7 | 0.00 - 17.30 |
| How many alcoholic drinks per week | 0 | 92.2 | 88.66 - 95.83 |
|  | 1 | 3.9 | 0.00 - 16.50 |
|  | 2 | 0.4 | 0.00 - 13.27 |
|  | 3 | 0.9 | 0.00 - 13.67 |
|  | 4 | 0.4 | 0.00 - 13.27 |
|  | 5 | 1.3 | 0.00 - 14.08 |
|  | 10 | 0.4 | 0.00 - 13.27 |
|  | 15 | 0.4 | 0.00 - 13.27 |
| How many days per week | 0 | 90.5 | 86.50 - 94.46 |
|  | 1 | 6.5 | 0.00 - 18.96 |
|  | 2 | 1.7 | 0.00 - 14.52 |
|  | 3 | 0.4 | 0.00 - 13.30 |
|  | 4 | 0.9 | 0.00 - 13.71 |
| Have you ever had a diagnostic of | Heart disease | 3.0 | 0.00 - 15.69 |
|  | High blood pressure | 9.9 | 0.00 - 22.13 |
|  | Stroke | 0.4 | 0.00 - 13.27 |
|  | Diabetes | 2.6 | 0.00 - 15.29 |
|  | Blood Clot | 0.4 | 0.00 - 13.27 |
|  | Asthma | 1.7 | 0.00 - 14.48 |
|  | Depression | 6.5 | 0.00 - 18.91 |
|  | Anxiety | 9.9 | 0.00 - 22.13 |
|  | PTSD | 4.3 | 0.00 - 16.90 |
|  | Chronic pain | 8.6 | 0.00 - 20.92 |
|  | None of the above | 73.3 | 66.62 - 79.93 |
| Have you ever been treated for | Heart disease | 2.2 | 0.00 - 14.88 |
|  | High blood pressure | 8.6 | 0.00 - 20.92 |
|  | Osteoarthritis | 3.0 | 0.00 - 15.69 |
|  | Osteoporosis | 2.6 | 0.00 - 15.29 |
|  | Depression | 5.2 | 0.00 - 17.70 |
|  | PTSD | 1.7 | 0.00 - 14.48 |
|  | Chronic pain | 9.5 | 0.00 - 21.73 |
|  | Anxiety | 7.3 | 0.00 - 19.71 |
|  | None of the above | 78.0 | 71.98 - 84.05 |
| BMI |  | 2,535.8 | 2,467.90 - 2,603.74 |
| BMI Class | Low | 1.3 | 0.00 - 14.14 |
|  | Normal | 55.2 | 46.57 - 63.87 |
|  | Overweight | 29.6 | 18.72 - 40.41 |
|  | Obese | 13.9 | 1.92 - 25.90 |
| IPAQ Class 1 | High | 58.6 | 50.34 - 66.90 |
|  | Moderate | 32.3 | 21.74 - 42.91 |
|  | Low | 9.1 | 0.00 - 21.32 |
| IPAQ Class 2 | Insufficient | 9.1 | 0.00 - 21.32 |
|  | Sufficient | 90.9 | 87.08 - 94.82 |
| SF-36 Female participants | No Limitation | 38.9 | 25.5 - 52.3 |
|  | Minor | 19.8 | 4.5 - 35.2 |
|  | Moderate/Severe | 41.2 | 28.1 - 54.4 |
| SF-36 Male participants | No Limitation | 50 | 36.1 – 63.9 |
|  | Minor | 16 | 0 - 34 |
|  | Moderate/Severe | 34 | 18.1 - 49.9 |
| K-10 Female participants | Well | 66.7 | 56.8 - 76.5 |
|  | Mild | 9.8 | 0 - 26 |
|  | Moderate/Severe | 23.5 | 8.6 - 38.4 |
| K-10 Male participants | Well | 76 | 66.4 - 85.6 |
|  | Mild | 11 | 0 - 29.5 |
|  | Moderate/Severe | 13 | 0 - 31.3 |
