# Supplementary material for: In search of a “vocabulary for recreation”: Leisure-time physical activity among humanitarian migrants in regional Australia
Source: PLoS One. 2020 Oct 14;15(10):e0239747. doi: 10.1371/journal.pone.0239747 (PMC7556461; doi:10.1371/journal.pone.0239747)
Supplement: Attachment — Submitted filename: Response to Reviewers.docx [file pone.0239747.s006.docx]

Ms. Re. No.: PONE-D-20-07177

In search of a “vocabulary for recreation”: Leisure-time physical activity among humanitarian migrants in regional Australia

Journal: PlosOne

|  | **Reviewer Comment** | **Response/Changes Made** |
| --- | --- | --- |
| **Reviewer #1** | At p. 3 lines 36-37, the authors claims that “In 2015, Australia received the third-largest number of refugees globally” while the cited data seem to refer only to the number of re-settled refugees. This is somehow very unclearly acknowledged at line 37, but this statement and the data mentioned would need much more clarity, as in fact UNHCR data (2016) showed that there were 21.3 million refugees globally in 2015. Such a clarification would be fundamental as the authors' claim about Australia's refugees reception seems to refer only to re-settled refugees, which only account for a very limited fraction of refugees globally. Secondly, clarifying that the numbers mentioned by the authors only refer to re-settled refugees, and not to refugees numbers overall, is important to avoid the unintended consequence of considering only re-settled refugees as “true” refugees worth considering in statistics. | We agree wholeheartedly with the reviewer that it’s important to make this clear beyond any questions. We have therefore added the word ‘re-settled’ before ‘refugees’ in the first sentence of the manuscript. |
|  | While employing the term “leisure” in the article from the title, the authors seem to have focused instead in the data collection to the participants' physical activities. As such, not much is known about other leisure practices that could be less directly implying physical activity, but can be defined as leisure (e.g. music-making, drama/theatre participation, sport fandom, arts, etc.), and could still be relevant to the authors' main focus on health. More elaboration would thus be needed as to why a specific focus on PA was chosen by the authors, but the arguably wider definition of leisure was used (instead of simply physical activity). There is a passing comment on this at p. 18, lines 422-424, but more elaboration is needed, and ideally much earlier in the paper (e.g. while setting out the research design and aims). | The reason for this focus on leisure-time physical activity is briefly explained in the first paragraph (original manuscript lines 40-45) but we agree that we need to make it a lot clearer and explicit for the reader and better justify the focus. A few changes were made throughout the paper to address this point, including on pages 2 (lines 38-42), 3 (lines 55-58), 5 (lines 113-116), 13 (lines 302-306) and 25 (lines 612-622) |
|  | Some contextual information would be relevant to add to address the specificities of the research context (e.g. to understand how such specificities played a role in influencing the participants leisure meanings and experiences), but also to shed more light on the research design process involved in the study. As such, some more elaboration on demographic, historical, and social domains of the context where the research was conducted would be needed. Furthermore, as the authors mention local “partners” more elaboration and contextual information would be important to explain how the collaboration started (e.g. how partners were involved in the project, who reached out to whom and why), what was the rationale for the collaboration across partners, how the community partners used (or aim to use) the research findings. This information could be relevant to provide important guidelines and references in terms of research design for scholars aiming to work with refugees and community organisations involved in supporting/advocating for refugees. | We have included an entire new section on Study Context, with more detailed information about the demographic and social context of the study, as well as a more information about our partners and their role in the project. The new material is in pages 6 and 7. |
|  | Sentence at p. 3 lines 40-46 too lengthy and unclear. Recommended to split it in two clearer and shorter sentences. | We have broken up the sentence into two clearer and shorter sentences (pg. 2, lines 38-44) |
|  | P. 6 Line 128 Why “mixed-methods research is a challenging area within which to work”? Clarify/elaborate or delete? | We have now deleted this sentence. |
|  | p. 8 line 133 “themes” possibly better wording than “names”? | This has been corrected. |
|  | P. 12 line 270, participant presented as “female from Sierra Leone”, while at line 273, same page, presented as “female from Liberia” | This has been corrected |
|  | P. 16, lines 380-381, “when you settle” could be worded better using the term “when refugees settle”, etc. | This has been corrected. |
|  | p. 17, lines 409-410 “our quantitative study did not find gender-based differences in participation, suggesting that other factors might be at play in this community” can the authors elaborate more on these “other factors” that might be at play, or indicate more clearly this as a further line of enquiry for research on the topic? | The sentence has been reworded to improve clarity of our point (page 20, lines 484-486) |
|  | P. 20 line 473 “PA” | This has been corrected. |
|  | P. 21 lines, 492-494 the authors state “Such a position is only recurrent in their narratives because it is equally entrenched in public discourse.” to what extent the data available justify this direct correlation between public discourses on leisure and the participants' narratives on leisure engagement? To what extent instead issues such as poverty, the “cold-panic” and ongoing worries for family and relationships elsewhere, and further factors play a role in making leisure to seem “impossible” for some of the participants? As these factors are considered in the paper's findings and discussion, it is likely just a matter for the authors to re-word the sentence above and allow for more openness in identifying factors at stake in what emerged from the data, consistently with their discussion overall. | We have now rephrased this sentence to state that “Such a position may be recurrent in their narratives because it is equally entrenched in public discourse” (page 23, lines 564-565) |
| **Reviewer #2** | Shorten the part of “Leisure Meanings” and summary key findings from the literature review at the end. | We have deleted two small sections of the Leisure Meanings section (approx. 150 words). |
|  | Give an overall description of the study design at the beginning of the method. How were the three stages of the study linked? Specify the (different) purposes of two qualitative studies (focus-group and in-depth interview) before and after the quantitative study. | We have now included more information about the relationship between each of the phases of the study in the Data Collection section, particularly for the focus groups (page 8, lines 173-177). Information about the purpose of interviews had already been provided (page 9, lines 209-211) |
|  | Kindly provide the interview guidelines for both the first and third studies, at least some key questions to help the reader understand the content. It might be better to provide backgrounds of the interviewers and interviewees as well. | We have now included as supplementary files the interview schedules for both focus groups and interviews and for the survey. |
|  | Any pilot testing of the interview guidelines and survey questionnaire? | No pilot testing was conducted. The instruments used in our survey are all well validated worldwide in different languages and populations, including refugees, and have been extensively used in scientific and governmental research and Census. For this reason, we decided to go without any pilot study and reliability assessment to use all the limited resources available in the actual data collection |
|  | Any information could be provided in terms of quality control (both the quantitative and qualitative parts)? | We have now added one paragraph in both Data Analyses sections providing information on quality control measures taken by the research team (page 10, lines 229-237 and 239-242). |
|  | For each quote, please provide the participant’s age, gender, and origin at the end in a bracket to provide supplemental information. | We have now added age details and moved the information to the end of the quotes as requested. |
|  | I noticed that (Line 215) most participants had no previous schooling (28.9%), although the majority were currently studying (51.9%). Is this low literacy affect the participants’ understanding of the study, particularly the second part of the survey that used validated scales? For example, SF-36 is quite long and complex. How was the questionnaire administrated? Was the questionnaire used in their original language? | A sentence was added in the Data Collection section clarifying that surveys were completed in English but that interpreters from the local community were available to provide assistance when needed (page 9, lines 205-207) |
|  | Line 233, I totally agree, “…further quantitative results are presented and discussed together with qualitative ones in order to facilitate integration of the knowledge gained from all phases of the study”. I have a few suggestions for the result presenting in this section: | |
|  | a) The current three themes were a bit general. Some keywords in these themes (“It Makes You Lazy”, A similar story”) were not specified. Details should be given, such as a similar story to what? | The theme names are purposely broad to encompass the diversity of experience of participants, while drawing their similarities. Two of the three themes are direct quotes from the participants, which we feel are important to acknowledge the participants’ voice in the study. We would prefer to keep them as they are now, although open to further discussing this with the reviewer and/or editor. |
|  | b) Is that possible to summarize a few subthemes under each theme? For example, In this context, participation in PA was seen as superfluous and a privilege they could not afford, both in terms of finance, time and also motivation. Here “finance”, “time”, and “motivation” and good subthemes to present. | Although we agree that in some cases there are some subthemes, this is not constant across the findings. By creating subthemes there is the potential that data become too fragmented and a less integrated picture of complexity and diversity is portrayed. Again, we’re happy to discuss this further with the reviewer and/or editor if required, but would prefer not to pursue this change to the manuscript. |
|  | c) Some sentences in the Results should be moved to the Discussion. For example, (Line 381) some of them are not too dissimilar to those experienced by other marginalised groups in our (Western) societies. Please present results derived from the present study only, and move potential explanations and comparisons (with others) to the Discussion. | The point of this statement cited by the reviewer is to contextualise the theme (i.e. A Similar Story) by showing that some challenges to participation in leisure are recurrent across population groups. Although we do discuss this point in more detail in the Discussion section, we feel it is important to leave this statement here precisely to provide an explanation for the theme |
|  | d) I did not see a clean message from the quantitative part; some key sentences would be useful. | Although some quantitative data are presented separately at the start of the Results section, results from the both quantitative and qualitative material were intended to be presented integrated. This is stated at the beginning of page 13:  “In the following sub-sections, further quantitative results are presented and discussed together with qualitative ones in order to facilitate integration of the knowledge gained from all phases of the study, where the rather “dry” numerical data can be nuanced and further explored for meanings and context. The themes from the qualitative phase of the study guide the organisation of the material, as they provide further depth to the results found in the quantitative phase of the study.”  We feel that providing summary statements only on the quantitative data would potentially compromise the flow of the argument and notion of integration of the different types of data. In addition, in the Discussion section, we have also addressed both qualitative and quantitative material combined. We would, therefore, prefer not to add any further summaries to avoid repetition and fragmentation of the material. |
|  | e) How about the validity of the SF-36 and K10 in the present study? | Given the two instruments are widely used internationally and have been validated to be used with different populations (including refugees in Australia), and also due to the challenges associated with recruitment of this marginalised population and our limited resources, we felt that it was not necessary to validate the instruments specifically for this study. |
|  | f) It might be better to move the two supplementary tables to the main paper because they provide essential information. | I am happy for the Journal Editor to make a decision on this one, given space limitations |
|  | Discuss a bit about the representativeness of the sample. Line 205 …”this sample represents approximately one third of the entire population”, this does not necessarily mean good representativeness. Any issues raised because of changing from respondent-driven sampling to convenience sampling in the second stage? | We have now included a more detailed discussion of the representativeness of the sample at the start of the Results section (page 11, lines 263-271) |
|  | Based on the findings presented in this study, what implications could be made to program officers or policymakers? | We have added a new section in the conclusion where we address implications for program officers and policymakers (page 26-27, lines 641-655), in addition to the last sentence of the conclusion (page 27, lines 658-661) |
|  | How could results derived from the present sample be generalized to other migrate populations worldwide? | We have now added more information on the generalizability of the findings (page 26-27, lines 641-655) |
|  | Discuss the limitations of the present study. | We have included two paragraphs discussing the limitations of the study at the end of the Discussion section (pages 25-26, lines 612-628) |
