# Supplementary material for: In search of a “vocabulary for recreation”: Leisure-time physical activity among humanitarian migrants in regional Australia
Source: PLoS One. 2020 Oct 14;15(10):e0239747. doi: 10.1371/journal.pone.0239747 (PMC7556461; doi:10.1371/journal.pone.0239747)
Supplement: Attachment — Submitted filename: Response to Reviewers.docx [file pone.0239747.s007.docx]

Ms. Re. No.: PONE-D-20-07177

In search of a “vocabulary for recreation”: Leisure-time physical activity among humanitarian migrants in regional Australia

Journal: PlosOne

|  | **Reviewer Comment** | **Response/Changes Made** |
| --- | --- | --- |
| **Reviewer #1** | The authors have addressed all the comments made in the previous review, and as a result the paper now offers a valid contribution to the topic it addresses.  I would only recommend one final, minor, yet substantial revision before recommending this paper for publication.  This relates to the automatic association of being a refugee with trauma that is often made in several points of the paper. Important contributions in the field of refugee studies have for some time challenged this automatic association, by convincingly arguing that, while it would be foolish to claim that displacement does not cause distress of many kinds, we cannot consider trauma as an axiom in relation to refugees' lives, as the implication of this association is to unwittingly expect these characteristics in this population (see Malkki, 1995, 1996; Fassin, 2005). These considerations point to avoid addressing refugees as “kind of persons” with shared and generalisable experiences, but as a broad, descriptive rubric that includes within it different socio-economic statuses, personal histories and psychological situations (see Malkki, 1995, p. 496). Despite being mentioned once by research participants in the data, “trauma” seems to have been used in the article a bit as a “catch-all” term in the paper and automatically associated with being a refugee. In other parts of the article, participants' experiences are qualified as traumatic despite seemingly addressing emotional and psychological experiences (e.g. ties and concerns for family members described as “baggage”) that would be misplaced to define as “trauma”. I would recommend caution in automatically associating trauma as an intrinsic qualifier of “being a refugee”, as these kind of automatic associations perpetuate assumptions and expectations of what a “real” refugee is that have some significant implications when these inform practice and policy. This is relevant as in the conclusions the authors discuss the generalisability of their findings exactly in terms of the “common experiences of trauma, etc.” (p. 28 line, 673 in the manuscript) can unwittingly contribute to normalise such problematic framings. While I understand this addition addressed one of Reviewer 2's comments, I would recommend shifting the core of this statement. From the present statement that argues generalisability despite the specificity of the research context, the authors could shift to one that argues generalisability because of shared challenges that refugee populations face in contexts of re-settlement (e.g. under-employment, limited social opportunities and mental health pressures, etc.) that would be important to understand in light of refugees' populations diversity and complexity. This relatively minor change would make a substantial difference in the level of nuance and contribution of this valid study to highlighting the relevance of leisure domains in contexts' of refugees' resettlement. | We fully agree and appreciate the reviewer’s comments. However, we would like to acknowledge that most, if not all, interview and focus group participants did share significant experiences of trauma, including witnessing murder, experiencing kidnapping and rape, literally losing loved ones in raids, walking for days across remote and inhospitable lands in search of safety, being forcibly removed from home leaving all possessions behind as well as other traumatic displacement experiences. Having listened to all of these stories certainly impacted on our writing, and the issue of trauma was, without doubt, present in our minds as we wrote this manuscript. We nonetheless agree that we cannot generalise these experiences, particularly across our survey participants, from whom we did not hear these stories. We therefore agree that we need to choose our words more carefully and therefore made subtle changes in four key locations, as follows:  1) Page 2, line 42: *However, it evolved to be an exploration of the meanings of leisure-time physical activity for ~~war and trauma survivors~~ refugees settled in a new and significantly different social and cultural environment, and how health is implicated in this process.*  2) Page 14, line 341: *A female participant, from Sierra Leone, described her struggles having to manage a busy life looking after children, studying to improve her English and work prospects, on top of a life of ~~trauma~~ displacement and worries*  3) Page 14, lines 356-358: *Some participants talked extensively about persistent pain and suffering ~~expanded on the issue of trauma~~, ~~but~~ particularly because of how they continue to deal with past experiences ~~it~~ vicariously through news of war and crime in their countries of origin*  4) Page 27, line 644: the discussion presented here may be clearly extrapolated to other refugee populations given the common, shared experience of displacement ~~trauma~~ and difficulties in adapting to and integrating into a new culture and society.  Other mentions of the word trauma were left unchanged as they were referring to findings and discussions from the literature. |

| **Additional changes** | As discussed with Dr Natasha McDonald, Associate Editor, we have moved the ethics statement back to the end of the Study Design sub-section in the Methods section to improve readability. |
| --- | --- |
