# Supplementary material for: In search of a “vocabulary for recreation”: Leisure-time physical activity among humanitarian migrants in regional Australia
Source: PLoS One. 2020 Oct 14;15(10):e0239747. doi: 10.1371/journal.pone.0239747 (PMC7556461; doi:10.1371/journal.pone.0239747)
Supplement: Attachment — Submitted filename: Response to Reviewers.docx [file pone.0239747.s008.docx]

Ms. Re. No.: PONE-D-20-07177

In search of a “vocabulary for recreation”: Leisure-time physical activity among humanitarian migrants in regional Australia

Journal: PlosOne

| **Associate Editor Comment** | **Response/Changes Made** |
| --- | --- |
| Study design needs to be framed within a framework like (happy for another to be used) CARE Or GRAMMS if mixed methods | Given the constructivist theory-approach adopted in this study, and following authors such as Buss & Perron (2020), Denzin & Lincoln (2018) and Hammersley (2008), we feel that such checklist frameworks are not always appropriate. As Buss & Perron argue, there is still controversy in the use of checklists to ascertain quality and rigour in qualitative research, particularly because of non-standardized research strategies and methodological pluralism, which we feel is the case here. The fact that this study moved from a positivist epistemological approach to a constructivist theory building exercise (as discussed in the next item below) means that non-standardised strategies were applied and therefore trying to fit them into standardised frameworks is fraught with problems. In addition, selecting one framework now to fit the request would require significant re-writing, which we feel will go against what previous reviewers have requested and also what they have praised in our study. We are happy, however, to provide further clarification on steps taken to ensure rigour in our analysis and we have done so in response to items listed below.  Buus, N., & Perron, A. (2020). The quality of quality criteria: Replicating the development of the consolidated criteria for reporting qualitative research (COREQ). International Journal of Nursing Studies, 102, 103452.  Denzin, N.K., Lincoln, Y.S. (Eds.) (2018). The SAGE Handbook of Qualitative Research. Sage, Thousand Oaks.  Hammersley, M. (2008). Questioning Qualitative Inquiry. Sage, London. |
| line 143 you state: “The methodology utilises positivist epistemology, a case study of Coffs Harbour, and constructivist theory-building” A positivist epistemology is Representational - People can know the reality and objectively explain it. Constructivism is an epistemology based on one’s own experiences built up through learning within a group or culture it is a subjectivist epistemology. – the statement could be confusing for the reader so please qualify or give context to stages | We have rephrased this sentence to provide more clarity in regards to our methodological approach. It now reads:  *The methodology utilises positivist epistemology, when focusing on a case study of Coffs Harbour and attempting to objectively assess the levels of engagement in leisure-time physical activity among refugee participants, and constructivist theory-building as a result of one participant's comment that there was no “vocabulary for recreation” available to these refugees [23]. This proved serendipitous in providing a framework upon which the project could be mounted and led the researchers to expand their methodological approach to embrace personal (subjective) understandings of experiences [23].* |
| Line 165-167 seems like results – can it be moved please | This line refers directly to the methodological approach utilised and is now better integrated in the explanation of the approach (quote in item above). |
| Line 171 – you consider stages and it seems like a mixed methods study so needs a name up front and given a type. | The statement that this is a mixed-method study was provided a few sentences before, at the start of the Study Design section. We have now clarified that it is a multistage mixed-methods study as per new wording presented below:  A case study approach was adopted with multi-stage mixed-methods being used for data collection [23]. |
| You need to justify your sample size across stages, e.g., you talk about saturation please give a type (data or theoretical) of saturation you refer to. Identify when saturation was identified in what number of interview? | We have added the following text regarding focus group sample size:  *Given this phase of the study was exploratory in nature and intended to inform the development of the survey, no data saturation was pursued and sample size was determined based on achieving a diverse representation of the local refugee population and ideal size of focus groups – research on focus groups has recommended that a minimum of four and a maximum of twelve participants be included in every one session to avoid silent voices and confusion, but still provide enough quorum to entice useful and productive discussions [25].*  We have also clarified that we used data saturation for interviews.  Below we explain issues related to sample size justification for the survey component of the study. |
| You need to give your power calculation for the survey | Power calculations in observational studies is a very unusual procedure, particularly when using convenience sapling. Although some efforts have been made to provide ways to estimate the sample size in advance (e.g. Hsieh, 1989; Maxwel, 2000), the power will depend heavily on the covariance between variables, which is hard to even guess, especially in a hard-to-reach population like that of refugees, with different backgrounds, and the estimation of the effect sizes. We acknowledge that some researchers try to use power calculations based on the univariate proportion of cases or an expected average. However, this approach is not adequate, especially due to the fact that our aim is not to simply estimate the level of physical activity, but mainly to investigate the variables associated with higher or lower levels of it. Also, even in attempts to develop a way to estimate power, most of the methods only present power related to a specific sample size (or the sample sizes associated with a specific power). Giving the non-linear nature of the procedure, it is not an easy task to extrapolate these values to different sample sizes or statistical power.  Hsieh, FY. Sample Size Tables for Logistic Regression. Statistics in Medicine, 8:795-802, 1989.  Maxwel, SE. Sample Size and Multiple Regression Analysis. Psychological Methods, 5(4):434-458, 2000. |
| You need to justify eligibility criteria for each stage | We have now included the following statement at the end of the Data Collection section:  *Eligibility criteria for participation in all stages of the study was broad and included any adult (i.e. 18 yrs or older) who self-identified as a resettled refugee member of the Coffs Coast community.* *The aim was to be as inclusive as possible of the diversity of members of this population.* |
| You need to give details of the development of the outcome measures used or interview schedules including piloting, cognitive interviews, development stages | Outcome measures utilised in the survey were all derived from validated surveys or developed from focus groups findings. This has been noted in the Data Collection section.  Given the exploratory nature of focus groups, questions were developed from the literature. A sentence to clarify this has now been added in the first paragraph of the Data Collection section.  There were no pilots or cognitive interviews conducted prior to formal data collection. Interviews were semi-structured and intended to explore issues identified in the survey responses, as described at the end of the Data Collection section. Therefore, the interview schedule was constructed without following pre-establish models/standards leaving also significant room for a free conversation to develop following participants’ narratives of experiences. |
| Audit trail to document qualitative analysis needs to be given | Although we appreciate the value of an audit trail, this process was not part of our data analysis procedures. Audit trails involve a person external to the study to review the data and data analysis processes to ensure the theoretical, methodological and analytic choices were valid and warranted (Morse, 2015; Liamputtong, 2020). We feel that we have provided the reasoning behind our choices in the paper to allow the reader, as an external party, to make judgements as to the quality of our processes. The Data Analysis section provides detailed description of the data analysis procedures, including measures to ensure rigour and trustworthiness, such as triangulation, thick description and lived experience. An audit trail documenting the qualitative analysis of the study is not a requirement for all qualitative studies but only one of several strategies that can be taken (Liamputtong, 2020; Denzin and Lincoln, 2018). It is also not something that is explicitly required for publication in PlosOne. We have added a sentence in the last paragraph of the Data Analysis section to further highlight the role of lived experience in the triangulation process.  Denzin, N.K., Lincoln, Y.S. (Eds.) (2018). The SAGE Handbook of Qualitative Research. Sage, Thousand Oaks.  Liamputtong, P. (2020). Qualitative Research Methods (5^th^ ed.). Oxford University Press.  Morse, J.M. (2015). Critical Analysis of Strategies for Determining Rigor in Qualitative Inquiry. Qualitative Health Research, 25(9): 1212-1222. |
| You must talk about the level of integration of data - fully or partial - if it is mixed methods? if there is no integration then its multi methods? | We have now included a new paragraph at the start of the Data Analysis section to clarify the data integration process. We changed the Qualitative Data Analysis and Quantitative Data Analysis sections to sub-sections within this new Data analysis section to make the processes a bit clearer too. The new paragraph now reads:  *Data analysis was fully integrated and occurred in various stages given the amount and complexity of the data. Below we explain the distinct processes for each type of data (i.e. quantitative and qualitative), but it is important to note that they did not occur in isolation. For instance, initial focus group exploratory analysis occurred prior to the development of the survey, and initial explanatory analysis of the surveys was conducted prior to interviews. However, analyses were re-visited and further theoretically explored once all data had been collected. This process is not uncommon in multistage mixed methods studies and provides a powerful strategy to achieve full integration of the material collected [29].* |
